# A Retrospective Cohort Study on HHV-8 Viral Load and Prognosis in HIV-Associated Kaposi Sarcoma Among People Living with HIV in Japan

**DOI:** 10.3390/v18020161

**Published:** 2026-01-25

**Authors:** K. Ishikawa, T. Muramatsu, S. Kaneko, Y. Harada, R. Miyashita, Y. Kamikubo, T. Yamaguchi, A. Ichiki, Y. Chikasawa, M. Bingo, R. Sekiya, M. Yotsumoto, T. Hagiwara, K. Amano, E. Kinai

**Affiliations:** 1 Department of Microbiology, Tokyo Medical University, Tokyo 160-8402, Japan; 2 Department of Infectious Diseases, Edogawa Hospital, Tokyo 133-0052, Japan; 3 Department of Laboratory Medicine, Tokyo Medical University Hospital, Tokyo 160-0023, Japan; tk4mrmz@tokyo-med.ac.jp (T.M.); mm401979@gmail.com (S.K.); celloyutan@gmail.com (Y.H.); lyui.miyashita@gmail.com (R.M.); yoshico0526@yahoo.co.jp (Y.K.); mt.mochi@gmail.com (T.Y.); too11young11to11die@gmail.com (A.I.); yushi_c@tokyo-med.ac.jp (Y.C.); bingo@tokyo-med.ac.jp (M.B.); ryosekiya@gmail.com (R.S.); mhk@tokyo-med.ac.jp (M.Y.); thagi@tokyo-med.ac.jp (T.H.); kamano@tokyo-med.ac.jp (K.A.); ekinai@tokyo-med.ac.jp (E.K.)

**Keywords:** HIV, AIDS, Kaposi sarcoma, Kaposi sarcoma inflammatory cytokine syndrome

## Abstract

Background: The characteristics and prognosis of HIV-associated Kaposi sarcoma (KS) among people living with HIV (PLWH), and their association with HHV-8 viral load are not well understood in Japan. Methods: We conducted a retrospective study of PLWH diagnosed with KS at Tokyo Medical University from 2000 to 2023. Results: Seventy cases of KS were identified; HHV-8 viral load data were available for twenty-three of these cases. The median age was 43 years (interquartile range [IQR], 11 years). The median HIV viral load at diagnosis was 150,000 copies/mL (IQR, 560,000 copies/mL). The median CD4 count was 76.0/μL (IQR, 157/μL). Lesions other than those of the skin were observed in the gastrointestinal tract (nine cases, 39.1%), oropharynx (three cases, 13.0%), and bronchial/lung (two cases, 8.7%). The median HHV-8 viral load was 0.0 copies/10^6^ WBC (IQR, 1500 copies/10^6^ WBC). Among the nine deceased PLWH, KS inflammatory cytokine syndrome (KICS) was diagnosed in five PLWH. Older age (≥50 years) and a high HHV-8 viral load (>615 copies/10^6^ WBCs) were significantly associated with worse survival. Conclusion: A high HHV-8 viral load may be a risk factor for mortality in PLWH with KS. Notably, all PLWH diagnosed with KICS in this study died, underscoring the poor prognosis associated with this condition.

## 1. Introduction

Kaposi sarcoma (KS) is an illness that defines acquired immunodeficiency syndrome (AIDS). KS is an angioproliferative disorder that requires infection with human herpesvirus 8 (HHV-8), also known as KS-associated herpesvirus, for its development [1,2]. KS causes lesions in the respiratory tract, gastrointestinal tract, and various internal organs, in addition to skin lesions.

Diseases associated with HHV-8, other than KS, include multicentric Castleman disease (MCD), germinotropic lymphoproliferative disorder, KS inflammatory cytokine syndrome (KICS), HHV-8-positive diffuse large B-cell lymphoma, primary effusion lymphoma (PEL), and extra-cavitary PEL [3]. The severity of KS is often associated with the presence of visceral KS or complications from other HHV-8-associated diseases.

KICS is a severe and urgent condition requiring rapid diagnosis and treatment, with established diagnostic criteria that include symptoms, clinical findings, inflammatory responses, and HHV-8 viral load [4]. Clinically, a relationship between HHV-8 plasma viral load and KICS is often observed. However, some retrospective studies have reported no significant association between HHV-8 viral load and KS, KICS, or MCD [5]. Additionally, KICS has been reported to occur regardless of CD4 count or human immunodeficiency virus (HIV) viral load. Previous studies have shown that at the time of KICS diagnosis, 80% of people living with HIV (PLWH) were already on antiretroviral therapy (ART), and in 50% of these PLWH, HIV viral load was suppressed [4].

The characteristics and prognosis of PLWH with KS at onset, as well as the complications of KICS, including HHV-8 viral load, are not well understood in Japan. Herein, we aimed to investigate the profiles of HIV-associated KS at diagnosis, assess subsequent clinical outcomes, and evaluate the association between HHV-8 serum viral load and prognosis.

## 2. Materials and Methods

### 2.1. Study Design and Setting

We conducted a retrospective analysis of PLWH medical records from the Department of Laboratory Medicine at Tokyo Medical University covering the years 2000 to 2023.

### 2.2. Ethics Statement

This study was approved by the Institutional Review Board of Tokyo Medical University, Tokyo, Japan (No.11000364). The requirement for participants consent was waived owing to the study’s retrospective nature.

### 2.3. Inclusion and Exclusion Criteria

We included adults (aged ≥ 18 years) living with HIV and diagnosed with biopsy-proven KS. Owing to the unavailability of KS-associated herpesvirus load measurements for all PLWH proven KICS could not be established in some cases (4). Consequently, we defined probable KICS criteria that do not require serum viral load measurements. HHV-8 serum viral load measurements were available for some PLWH with KS, performed using the GenIQ HHV-8 test (BML, Inc., Tokyo, Japan). HHV-8 serum viral loads below 20 copies/10^6^ white blood cells (WBC) were treated as 0 copies/10^6^ WBC, and values equal to or exceeding 1.0 × 10^7^ copies/10^6^ WBC were considered outliers and excluded from the analysis.

### 2.4. Study Definition and Outcome

The primary outcome of the study was all-cause mortality among PLWH with KS. We also analyzed the baseline characteristics of the PLWH with KS and evaluated treatment-related adverse events, the prognosis of KS, and the association of HHV-8 viral load with prognosis. To evaluate potential selection bias between all PLWHwith KS and those with HHV-8 viral load data, we compared the characteristics of both groups.

We defined aviremic AIDS-KS as KS occurring in PLWH taking ART with a HIV viral load less than 50 copies/mL for more than 12 months [6]. Adverse events due to chemotherapy were evaluated using the Common Terminology Criteria for Adverse Events v5.0.

### 2.5. Statistical Analysis

Categorical variables were compared using the chi-square test. Continuous variables were analyzed using the Mann–Whitney U test for non-normally distributed data or Student’s t-test for normally distributed data. Due to the retrospective nature of this study, patients were categorized into two groups for comparison: the HHV-8 measurement group and the non-measurement group, in order to estimate potential selection bias. The measurement group comprised patients for whom HHV-8 viral load was assessed, regardless of whether the results were above or below the detection limit. The non-measurement group consisted of individuals for whom HHV-8 testing was not performed. Standardized mean differences (SMDs) were utilized to evaluate clinical imbalances between these two cohorts, ensuring a robust assessment of baseline characteristics regardless of the availability of HHV-8 data.

To evaluate prognostic factors for mortality, Kaplan–Meier survival curves were generated, and differences between groups were assessed using the log-rank test. PLWH were stratified based on pre-defined, clinically relevant cutoffs. These included age (cutoff: 50 years), CD4 count (cutoff: 200 cells/µL), and HIV viral load (cutoff: 50 copies/mL) to assess the impact of immunodeficiency and virologic control.

For the HHV-8 viral load, several exploratory analyses were performed to investigate its prognostic value using different thresholds: (i) a cutoff of >100 copies/10^6^ WBCs, based on a previous report on KICS (4); (ii) the presence of viremia (detectable vs. undetectable), a concept applied in studies on aviremic KS [6]; and (iii) an optimal cutoff value derived from receiver operating characteristic (ROC) curve analysis to discriminate between survivors and non-survivors.

Multivariate analysis was conducted using the Cox proportional hazards model. To evaluate the impact of HHV-8 viral load on mortality while adjusting for potential confounders, hazard ratios and 95% confidence intervals were calculated using the same model. A *p*-value < 0.05 was considered statistically significant. All analyses other than SMD were performed using SPSS Statistics version 29.01J (IBM Japan, Tokyo, Japan). All statistical analyses and SMD computations were performed using R version 4.5.0 (11 April 2025; The R Foundation for Statistical Computing, Vienna, Austria).

## 3. Results

### 3.1. Participants’ Characteristics and Selection

**Figure 1:**
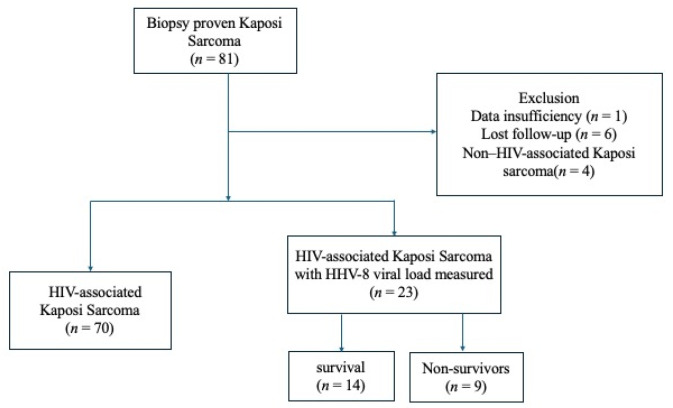
Flowchart of participants selection and classification in this study. Abbreviation: HIV, human immunodeficiency virus.

Among 81 with biopsy-proven KS, one case was excluded due to insufficient data, six were lost to follow-up, and four were identified as non–HIV-associated KS, resulting in 70 cases of HIV-associated KS. All patients were Japanese. Among these 70 PLWH, HHV-8 viral load was measured in 23 cases (Figure 1). The analysis of HHV-8-related prognostic indicators included 14 survivors and 9 non-survivors.

### 3.2. Comparison of Participant Cohorts

In Table 1, most baseline clinical characteristics were well-balanced; however, the measurement group showed a higher prevalence of gastrointestinal involvement (SMD = 1.06), smoking (SMD = 0.513), and a higher rate of systemic chemotherapy (SMD = 0.531). Regarding clinical outcomes, the measurement group was characterized by a significantly higher mortality rate (39.1% vs. 2.1%; SMD = 1.028) and a shorter follow-up duration (SMD = −1.520) compared to the non-measurement group. These findings suggest that HHV-8 viral load tended to be measured in patients with more severe clinical courses.

### 3.3. Treatment and Clinical Course of Unique Cases

Two cases developed after long-term initiation of ART at 7778 and 5866 days, respectively. A total of 35 of the 70 PLWH with KS received PLD. The number of treatment courses ranged widely: while many patients received only a few cycles, one individual underwent up to 47 courses, exceeding the recommended lifetime dose of 550 mg/m^2^ with informed consent. Cardiotoxicity was carefully monitored throughout treatment, and no PLD-associated cardiotoxic events were observed [7]. One patient received second-line chemotherapy with paclitaxel (three cycles) before switching to PLD, completing 30 cycles in total.

### 3.4. Prognostic Factors and Outcomes

**Figure 2:**
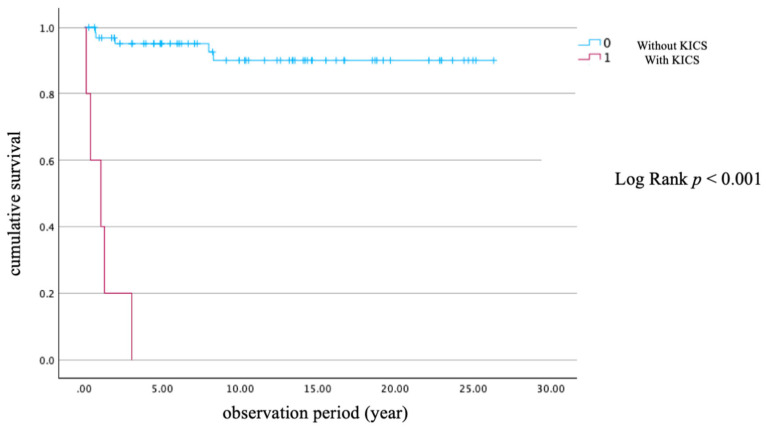
Kaplan–Meier survival curve with and without KICS. Abbreviation: KICS, Kaposi sarcoma inflammatory cytokine syndrome.

Table 2 summarizes the baseline characteristics and outcomes of 23 PLWH with KS, comparing those who survived (*n* = 14) with those who died from KS (*n* = 9). Survivors had a significantly lower age (36.0 years, IQR 14) compared to non-survivors (46.0 years, IQR 9; *p* = 0.003). HIV viral load was significantly higher among survivors (280,000 copies/mL, IQR 580,500) compared to non-survivors (62,000 copies/mL, IQR 131,000; *p* = 0.023). Although the CD4 count and HHV-8 viral load did not differ significantly (*p* = 0.600 and 0.124, respectively), the median number of days from ART initiation to KS diagnosis was significantly longer in non-survivors (10.0 days, IQR 177) than in survivors (–21.0 days, IQR 45; *p* = 0.046), suggesting that KS was more often diagnosed prior to ART administration in the survival group. KICS was observed in five of nine non-survivors (55.6%) and none of the survivors (*p* = 0.002), indicating its strong association with poor prognosis. The median follow-up duration was significantly longer in survivors (4.32 years, IQR 6.86) than in non-survivors (1.04 years, IQR 1.95; *p* = 0.028). No significant differences were found in sex distribution (all PLWH in this subgroup were male), smoking, alcohol use, site of lesion, chemotherapy administration, or the proportion of PLWH with KS onset prior to ART initiation. Figure 2 presents a Kaplan–Meier survival curve stratified by the presence or absence of KICS. PLWH with KICS had a significantly shorter survival time (log-rank test *p* < 0.001), ranging from 0.096 to 3.02 years.

### 3.5. Survival Analysis Using Pre-Defined Clinical Cutoffs

**Figure 3:**
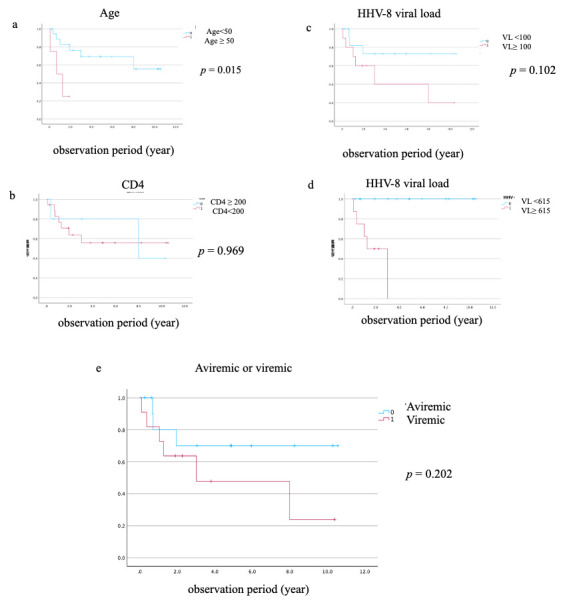
Kaplan–Meier survival curves for all-cause mortality. Kaplan–Meier curves comparing the survival of PLWH with HIV-associated Kaposi sarcoma (*n* = 23) stratified by (**a**) age (<50 vs. ≥50 years), (**b**) CD4 count at diagnosis (<200 vs. ≥200 cells/µL), (**c**) a low HHV-8 viral load cutoff (≥100 copies/10^6^ WBCs), (**d**) a high HHV-8 viral load cutoff (≥615 copies/10^6^ WBCs), and (**e**) the presence of HHV-8 viremia. *p*-values were calculated using the log-rank test.

**Figure 4:**
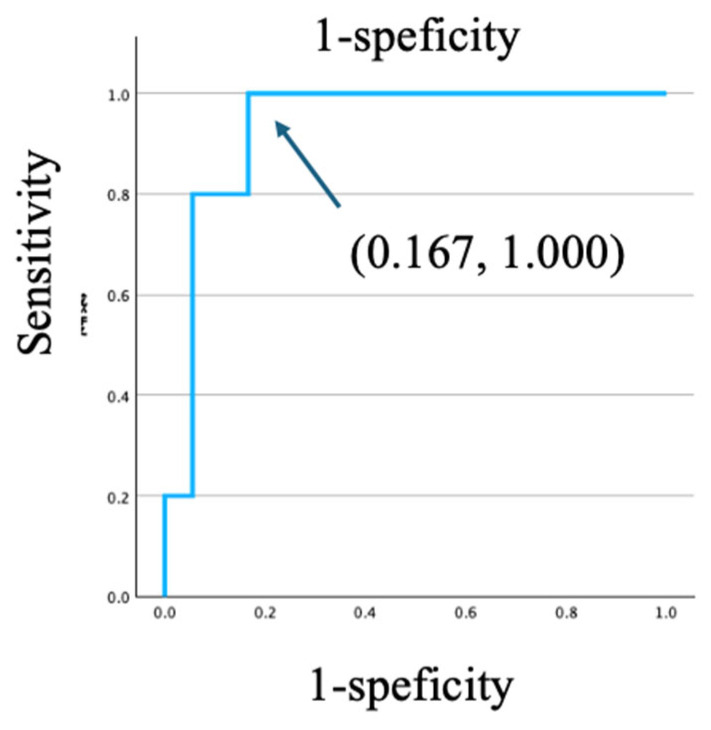
ROC curve analyses for predictors of KICS-related death for HHV-8 viral load.

To identify prognostic factors for all-cause mortality, we conducted Kaplan–Meier survival analyses using pre-defined, clinically relevant cutoffs. The analysis revealed that age ≥50 years was significantly associated with worse survival (log-rank *p* = 0.015) (Figure 3a). HHV-8 viral load demonstrated strong discriminatory ability for mortality, with a cutoff value of 615 copies/10^6^ WBC, yielding a sensitivity of 1.000 and a specificity of 0.833 (AUC = 0.933, Figure 4). Similarly, a high HHV-8 viral load (>615 copies/10^6^ WBCs) was a strong predictor of mortality (*p* = 0.026) (Figure 3d). In contrast, there were no significant differences in survival when PLWH were stratified by CD4 count < 200 cells/µL (*p* = 0.969), HHV-8 viral load > 100 copies/10^6^ WBCs (*p* = 0.102), or the presence of HHV-8 viremia (*p* = 0.202) (Figure 3b,c,e). A survival analysis based on HIV viral load (>50 copies/mL) could not be performed, as all PLWH in the non-survivor group had a viral load above this threshold. The Cox proportional hazards model was used to evaluate the association between HHV-8 viral load and mortality, adjusting for clinically relevant variables including age, CD4 count, HIV viral load, and the presence of KICS. In the multivariate Cox proportional hazards model, none of the variables maintained statistical significance (*p* < 0.05). The independent effects of baseline factors, including HHV-8 status and KICS, were attenuated in the adjusted model, likely due to the limited number of death events (*n* = 10). This resulted in insufficient statistical power and wide 95% confidence intervals for the predictors.

### 3.6. Clinical Characteristics of Participants with KICS

Table 3 summarizes the clinical characteristics of five PLWH diagnosed with KICS. All five ultimately died. The median age was 46 years (range 43–55), and the median initial CD4 count was 93/µL (range 13–460). The median initial HIV viral load was 26,000 copies/mL (range 1300–50,000), and the median HHV-8 viral load was 25,000/10^6^ WBC (1000 to >1.0 × 10^7^ copies). Three received KICS-specific treatment: one received pegylated liposomal doxorubicin in combination with tocilizumab, one received tocilizumab alone, and one received etoposide. Two received no specific KICS treatment. The median duration from KICS diagnosis to death was 12.5 months (range 1.15–36.2).

## 4. Discussion

To our knowledge, this is the first retrospective study to investigate the characteristics and outcomes of HIV-associated KS in a Japanese PLWH cohort. Our survival analysis identified that older age (≥50 years) and a high HHV-8 viral load (>615 copies/10^6^ WBCs) are significant factors for a poor prognosis in the univariate analysis. These findings contribute to a deeper understanding of KS in the modern ART era.

Historically, KS was a classic AIDS-defining illness, typically occurring in ART-naïve individuals with profound immunosuppression [8]. Before the widespread availability of ART, the risk of developing KS in PLWH was extremely high, a situation driven by the transmission dynamics of its etiologic agent, HHV-8. A large-scale cohort study in the pre-ART era demonstrated that for individuals co-infected with HIV and HHV-8, the 10-year probability of developing KS was approximately 50% [2]. The advent of potent ART has fundamentally altered this landscape. Current clinical guidelines from the U.S. Department of Health and Human Services and the World Health Organization recommend ART for all PLWH with HIV-associated KS, as it forms the cornerstone of management [8,9]. By suppressing HIV replication and enabling immune recovery, ART has dramatically reduced the incidence of KS, transforming its natural history. However, this process of immune recovery has also introduced a new clinical challenge: immune reconstitution inflammatory syndrome (IRIS). KS-IRIS can manifest as either “unmasking” of subclinical disease or “paradoxical” worsening of preexisting lesions, typically occurring within months of starting ART. Risk factors for KS-IRIS include advanced tumor stage, high pre-ART HIV viral load, and detectable plasma HHV-8 DNA, highlighting the role of antigen burden in its development [10].

Beyond the context of IRIS, KS can also develop or recur in PLWH on long-term ART with sustained HIV virologic suppression, a condition sometimes termed ‘aviremic KS’ [6,11]. A comparative study observed that PLWH with aviremic KS, similar to those with classic KS, may have a more indolent clinical course compared to those with viremic HIV-associated KS [6]. Although the precise mechanisms for this remain under investigation, hypotheses include incomplete restoration of HHV-8-specific T-cell immunity, chronic inflammation, and local immune exhaustion within tissues [12]. These diverse clinical presentations and proposed mechanisms suggest that KS pathogenesis in the modern ART era is multifactorial and cannot be explained solely by systemic CD4 cell counts [13].

In our study, survival analysis demonstrated that the presence of KICS was a powerful predictor of mortality, and a high HHV-8 viral load (≥615 copies/10^6^ WBCs) was significantly associated with a poor prognosis. These two findings are intrinsically linked, as all five PLWH who died from KICS exhibited markedly high HHV-8 viral loads. This suggests that the prognostic power of a high viral load in our cohort is largely driven by its ability to identify PLWH with this lethal syndrome. This aligns with the established definition of KICS as a syndrome characterized by severe inflammatory symptoms and, critically, elevated HHV-8 viremia [4]. A limitation of our study is that we did not assess cytokine levels, such as IL-6. However, the lethality of KICS is driven by a cytokine storm stimulated by this intense viral activity, with levels of hIL-6, vIL-6, and IL-10 comparable to those in active KSHV-MCD [4,10,13,14].

Therefore, the high HHV-8 viral load observed in our deceased PLWH should be interpreted as a direct reflection of the underlying lethal pathophysiology of KICS. Among the five participants with HHV-8 viral load ≥615 copies/10^6^ WBC, three survived. Two of these survivors received PLD (two and six cycles, respectively), both demonstrating clinical improvement without requiring second-line chemotherapy. Although limited by small numbers, these findings suggest that even patients with markedly elevated HHV-8 viremia may respond favorably to PLD, in some cases with only a relatively small number of treatment cycles.

Regarding lesion sites, our findings that the gastrointestinal tract, oropharynx, and lungs were common sites of involvement are consistent with previous findings [15,16,17]. The consistency across studies underscores the clinical importance of screening for KS in these specific organ systems. In our study, a clear association between the presence of visceral lesions and mortality was not observed, which may be attributable to the limited number of PLWH with such involvement in our cohort. However, this should be interpreted with caution. It is widely established in the literature that visceral involvement, particularly of the lungs and gastrointestinal tract, is a significant poor prognostic factor in PLWH with KS. Nevertheless, comprehensive reviews and studies from the modern ART era indicate that outcomes for PLWH with visceral KS, including gastrointestinal tract involvement, have substantially improved with the combination of effective ART and, when indicated, systemic chemotherapy [16,17]. Therefore, although our study did not have the statistical power to demonstrate this effect, the existing literature underscores the critical importance of screening for and appropriately managing visceral disease to improve overall survival in PLWH with KS.

KS mortality has dramatically changed over time but remains a concern. Mortality rates vary significantly by geographic region [18,19] and even by racial and ethnic groups within the same country, suggesting the influence of healthcare access and socioeconomic factors [20]. Furthermore, the HHV-8 genotype varies between countries, which may influence the outcomes, although this was not explored in our study [21].

Although no standard therapy for KICS has been established, rituximab monotherapy [13] or combination therapy with liposomal doxorubicin and rituximab has shown potential efficacy [14]. In our study, only one of the five PLWH with KICS received liposomal doxorubicin in combination with tocilizumab; the remaining PLWH were treated with tocilizumab alone, etoposide, or no specific treatment.

This study has some limitations. It is a retrospective study; hence, we cannot rule out missing data and selection bias in PLWH whose HHV-8 viral loads were measured. There were no significant differences in background factors regardless of whether HHV-8 was measured.

The HHV-8 viral load was measured only at the time of KS diagnosis and was not monitored during chemotherapy, because repeat testing is not covered by the national health-insurance system in Japan. As a result, we were unable to evaluate longitudinal changes in HHV-8 viremia or determine whether reductions in viral load correlate with treatment response and prognosis. Although the clinical significance of dynamic HHV-8 kinetics remains uncertain, this limitation prevents us from drawing conclusions regarding the relationship between therapeutic effect and virologic improvement. Future studies incorporating serial viral-load measurements will be essential to address this gap.

The statistical power to detect significant effects was limited by the small sample size. Additionally, although the long study period may allow for the observation of a link between ART to KS, we could not track this in the study.

Furthermore, KICS is a rare but highly lethal complication of HIV-associated Kaposi sarcoma, making it inherently difficult to evaluate its prognostic factors in single-center studies. Large-scale international collaborative studies will therefore be essential to validate the prognostic role of HHV-8 viral load, to account for regional and virological heterogeneity, and to establish more robust evidence to guide the management and improve outcomes of PLWH with KS and KICS.

## 5. Conclusions

In conclusion, despite undergoing chemotherapy, PLWH with KS had a high mortality rate, and all PLWH who developed KICS died. Our study suggests that KS can occur regardless of CD4 count and the duration of ART. In cases that developed KICS, the HHV-8 viral load was higher, and since all KICS died from KICS-related causes, a relationship between HHV-8 viral load and prognosis was indicated. Further research is needed to investigate confounding factors and validate these findings.

## Figures and Tables

**Table 1:** Comparison of baseline characteristics and outcomes between HHV-8 measurement and non-measurement groups.

	KS (*n* = 70)	KS with HHV-8 Viral Load Measurement (*n* = 23)	KS Without HHV-8 Viral Load Measurement (*n* = 47)	SMD
Median age (interquartile range: IQR)	42 (14)	43 (11)	42 (14)	−0.081
Male, *n* (%)	70 (100)	23 (100)	47 (100)	
Smoking status, *n* (%)	34 (48.6)	15 (65.2)	19 (40.4)	0.513
Alcohol use, *n* (%)	6 (8.6)	1 (4.3)	5 (10.6)	−0.241
HIV viral load (copies/mL) (IQR) (minimum–maximum)	1.5 × 10^5^ (2.975 × 10^5^) (2.4 × 10–4.3 × 10^6^)	1.5 × 10^5^ (5.6 × 10^5^) (1.3 × 10^3^–1.4 × 10^6^)	1.6 × 10^5^ (2.9 × 10^5^) (2.4 × 10 – 4.3 × 10^6^)	0.076
CD4 count (/µL) (IQR) (minimum–maximum)	69 (175.7) (4–1121)	76.0 (157) (4–566)	55.7 (194) (5–1121)	−0.131
HHV-8 viral load (copies/10^6^ WBC) (IQR)	0.0 (1.5 × 10^3^)	0.0 (1.5 × 10^3^)	N/A	N/A
site of lesion				
Skin, *n* (%)	51 (72.9)	17 (73.9)	34 (72.3)	0.035
Gastrointestinal, *n* (%)	25 (35.7)	9 (39.1)	16 (34.0)	1.06
Oropharynx, *n* (%)	10 (14.3)	3 (13.0)	7 (14.9)	−0.053
Lung, bronchial, *n* (%)	4 (5.7)	2 (8.7)	2 (4.3)	0.181
Others, *n* (%)	7 (10.0)	4 (17.4)	3 (6.4)	0.345
Chemotherapy, *n* (%)	40 (57.1)	17 (73.9)	23 (48.9)	0.531
Pegylated liposomal doxorubicin (PLD), *n* (%)	35 (87.5)	16 (94.1)	19 (82.6)	
Median number of PLD (IQR) (cycle)	3 (4), maximum of 47	3 (15), maximum of 47	3 (4), maximum of 22	
GRADE 3 adverse events, *n* (%)	0 (0)	0 (0)	0 (0)	
Three courses of paclitaxel following doxorubicin, *n* (%)	1 (2.5)	1 (4.3)	0 (0)	
ABVD, *n* (%)	1 (2.5)	0 (0)	1 (4.3)	
ABV, *n* (%) with Hodgkin lymphoma	1 (2.5)	0 (0)	1 (4.3)	
R-EPOCH, *n* (%) with DLBCL	1 (2.5)	0 (0)	1 (4.3)	
KS onset before ART, *n* (%)	43 (61.4)	14 (60.9)	29 (61.7)	−0.017
Median day from the start of ART to the diagnosis of KS, *n* (%) (minimum–maximum)	−12.50 (56) (−64–7778)	−11.0 (53) (−55–387)	−13.5 (57) (−64–7778)	−0.232
Aviremic cases, *n* (%)	1 (1.43)	0 (0)	1 (2.1)	−0.211
KICS, *n* (%)	5 (7.1%)	5 (21.7)	0 (0)	0.745
Mortality, *n* (%)	10 (14.3)	9 (39.1)	1 (2.1)	1.028
KICS	5 (50%)	5 (55.6)	0 (0)	
Progressive multifocal leukoencephalopathy	2 (20%)	2 (22.2)	0 (0)	
Pancreatic cancer	2 (20%)	1 (11.1)	1 (100)	
Median follow-up year (IQR)	8.7 (12.7)	2.3 (5.22)	13.45 (12.2)	−1.52

Abbreviation: KS, Kaposi sarcoma; HIV, human immunodeficiency virus; ART, antiretroviral therapy, KICS, Kaposi sarcoma inflammatory cytokine syndrome; DLBCL, diffuse large B cell lymphoma; SMD, Standardized Mean Difference calculated by Cohen’s d.

**Table 2:** Baseline characteristics and outcomes of patients with HIV-associated Kaposi sarcoma by survival status.

	Survival (*n* = 14)	Non-Survivors (*n* = 9)	*p*
Median age (IQR)	36.0 (14)	46.0 (9)	**0.003**
Male, *n* (%)	14 (100)	9 (100)	
Smoking status, *n* (%)	9 (64.3)	6 (66.7)	0.907
Alcohol use, *n* (%)	1 (7.1)	0 (0.0)	0.412
HIV viral load (copies/mL) (IQR) (minimum–maximum)	280,000 (580,500)	62,000 (131,000)	**0.023**
CD4 count (/µL) (IQR) (minimum–maximum)	90.8 (150.2)	70.0 (264.3)	0.600
HHV-8 viral load (copies/10^6^ WBC) (IQR)	0.0 (547.5)	1000 (29,000)	0.124
Site of lesion			
Skin, *n* (%)	11 (78.6)	6 (66.7)	0.526
Gastrointestinal, *n* (%)	7 (50.0)	2 (22.2)	0.183
Oropharynx, *n* (%)	3 (21.4)	0 (0.0)	0.136
Lung, bronchial, *n* (%)	0 (0.0)	2 (22.2)	0.065
Other, *n* (%)	2 (14.3)	2 (22.2)	0.624
Chemotherapy, *n* (%)	9 (64.3)	8 (88.9)	0.190
Pegylated liposomal doxorubicin *n* (%)	8 (57.1)	8 (88.9)	0.106
KS onset before ART, *n* (%)	10 (71.4)	4 (44.4)	0.196
Median day from the start of ART to the diagnosis of KS, *n* (%) (minimum–maximum)	−21.0 (45)	10.0 (177)	**0.046**
aviremic cases, *n* (%)	0 (0.0)	0 (0.0)	
KICS, *n* (%)	0 (0.0)	5 (55.6)	**0.002**
Median follow-up year (IQR)	4.32 (6.86)	1.04 (1.95)	**0.028**

Abbreviation: IQR, interquartile range; KS, Kaposi sarcoma; HIV, human immunodeficiency virus; ART, antiretroviral therapy; KICS, Kaposi sarcoma inflammatory cytokine syndrome; WBC, white blood cell.

**Table 3:** Clinical characteristics and outcomes of patients with KICS.

Case	Age	Initial CD4 Count (/µL)	Initial HIV Viral Load (Copies/mL)	HHV-8 Viral Load (Copies/10^6^ WBC)	KICS-Specific Treatment	Months from KICS Diagnosis to Death
Case 1	50	17	150,000	>1.0 × 10^7^	Doxorubicin + Tocilizumab	4.46
Case 2	46	460	25,000	25,000	Tocilizumab	36.2
Case 3	43	114	90,000	33,000	Etoposide	12.5
Case 4	45	13	26,000	1000	None	1.15
Case 5	55	93	1300	11,000	None	15.2

Abbreviation: KS, Kaposi sarcoma; HIV, human immunodeficiency virus; ART, antiretroviral therapy, KICS, Kaposi sarcoma inflammatory cytokine syndrome; DLBCL, diffuse large B cell lymphoma.

## Data Availability

The data presented in this study are available from the corresponding author (K.I.) upon reasonable request.
